# Supplementary material for: Homologous Recombination Deficiency (HRD) and BRCA 1/2 Gene Mutation for Predicting the Effect of Platinum-Based Neoadjuvant Chemotherapy of Early-Stage Triple-Negative Breast Cancer (TNBC): A Systematic Review and Meta-Analysis
Source: J Pers Med. 2022 Feb 21;12(2):323. doi: 10.3390/jpm12020323 (PMC8876589; doi:10.3390/jpm12020323)
Supplement: Supplementary file 1 [file jpm-12-00323-s001.zip › jpm-1557398-supplementary.pdf]

Supplemental Table S1. The definitions of pCR in the included studies.

Author	Study	Year	the definition of pCR
Fasching et al.[19]	GeparPLA	2020	pCR was defined as no residual invasive tumor in breast and in axillary lymph nodes (ypT0/is ypN0).
Mayer et al.[20]	TBCRC030	2020	No detailed description.
Yuan et al.[21]	NCT01525966	2020	pCR was defined as no residual invasive breast cancer with or without DCIS in the breast and axilla (ypT0/TisN0).
Sharma et al.[22]	NeoSTOP	2020	pCR was defined as no residual invasive breast cancer with or without DCIS in the breast and axilla (ypT0/TisN0).
Fontaine et al.[23]	BSMO	2019	pCR was defined as no residual invasive tumor in breast and in axillary lymph nodes (ypT0/is ypN0).
Hahnen et al.[15], Loibl.S et al.[25]	GeparSixto	2017, 2018	pCR was defined as either no invasive residuals in breast and lymph nodes (ypT0/is ypN0) or no invasive and no non-invasive residuals in breast and lymph nodes (ypT0 ypN0).
Loibl et al.[26], Telli et al.[27]	BrighTNess	2018	pCR was defined as no residual invasive disease on evaluation of the resected breast specimen and resected lymph nodes (ie, ypT0/is ypN0).
Sella et al.[32]		2018	pCR was defined as no residual invasive disease (with or without DICS) in the breast and no any tumor deposit> 0.2 mm in sampled axillary nodes (ypT0/isN0).
Sharma et al.[28]	PROGECT	2017	PCR was defined as no residual invasive disease with or without DICS in the breast and axilla (ypT0/isN0).
Connolly et al.[29]	TBCRC 008	2016	PCR was defined as no invasive cancer in breast/axilla.
Telli et al.[30]	PrECOG 0105	2015	PCR was defined as no invasive carcinoma in the breast and axillary lymph nodes.
Kaklamani et al.[31]	NCT01372579	2015	pCR was defined as no histologic evidence of invasive tumor cells in the surgical breast specimen and axillary lymph nodes.
Silver et al.[23]		2010	PCR was defined as a score of 5 according to the Miller-Payne scoring system.

Abbreviations: pCR: pathological complete response CR: DCIS: ductal carcinoma in situ.
